# Redefinition of the Mora Romagnola Pig Breed Herd Book Standard Based on DNA Markers Useful to Authenticate Its “Mono-Breed” Products: An Example of Sustainable Conservation of a Livestock Genetic Resource

**DOI:** 10.3390/ani11020526

**Published:** 2021-02-18

**Authors:** Silvia Tinarelli, Anisa Ribani, Valerio Joe Utzeri, Valeria Taurisano, Claudio Bovo, Stefania Dall’Olio, Francesco Nen, Samuele Bovo, Giuseppina Schiavo, Maurizio Gallo, Luca Fontanesi

**Affiliations:** 1 Department of Agricultural and Food Sciences, Division of Animal Sciences, University of Bologna, Viale Fanin 46, 40127 Bologna, Italy; silvia.tinarelli2@unibo.it (S.T.); anisa.ribani2@unibo.it (A.R.); valeriojoe.utzeri2@unibo.it (V.J.U.); valeria.taurisano2@unibo.it (V.T.); stefania.dallolio@unibo.it (S.D.); samuele.bovo@unibo.it (S.B.); giuseppina.schiavo2@unibo.it (G.S.); 2 Associazione Nazionale Allevatori Suini, Via Nizza 53, 00198 Roma, Italy; f.nen@anas.it (F.N.); gallo@anas.it (M.G.); 3 Associazione Regionale Allevatori dell’Emilia-Romagna, Viale Della Mercanzia 240-242-244, 40050 Funo di Argelato (BO), Italy; direzione@araer.it

**Keywords:** authenticity, autochthonous breed, conservation genetics, fraud, genetic resources, SNP, *Sus scrofa*

## Abstract

**Simple Summary:**

Autochthonous breeds are, in general, well adapted to their production systems in which they have been constituted but they are usually less efficient than commercial breeds. Therefore, conservation strategies of livestock genetic resources should be designed to assure profitability to the farmers. The development of “mono-breed” brand products is one of the most effective actions towards this aim. These products are usually sold at a higher price compared to undifferentiated ones, as the consumers consider positively the link between these breeds and the perceived quality of their products. The premium price, however, also attracts fraudsters that unscrupulously see an economic advantage by selling mis-labelled products to obtain an unjustified additional economic gain. These frauds undermine the whole strategy designed to support a sustainable conservation of autochthonous genetic resources. Mora Romagnola is a local pig breed raised in the north of Italy. Mono-breed pork products derived from this breed are part of an important niche value chain that is intrinsically linked to the conservation of this local genetic resource. In this study we present how the Mora Romagnola Herd Book standard integrated information of DNA markers of two genes (*MC1R* and *NR6A1*), affecting morphological traits, to allow the authentication of mono-breed products of this breed. This is one of the first examples of sustainable conservation of a pig genetic resource designed starting from the genotype of the animals registered to the breed herd book, with the specific purpose to combat frauds.

**Abstract:**

Mora Romagnola is an autochthonous pig breed, raised in the north of Italy. Mono-breed pork products of this breed are part of important niche value chain that is intrinsically linked to the conservation of this local genetic resources that can only survive due to the premium price that these products can obtain on the market. However, the added value attracts fraudsters that unscrupulously sell mis-labelled Mora Romagnola products, causing consumer distrust that, in turn, undermines the conservation strategy of this breed. To monitor and better characterise this local breed, we phenotyped 826 Mora Romagnola pigs for three breed-specific traits. Then, we genotyped almost all living sows and boars registered to the Herd Book (n. = 357 animals) for polymorphisms in the *MC1R* and *NR6A1* genes (affecting coat colour and vertebral number, respectively). The results were used to re-define the breed descriptors of the Mora Romagnala breed that included information on the allowed genotypes at these two genes. A few pigs that did not carry the allowed genotypes were excluded from its Herd Book. Finally, we evaluated the usefulness of these DNA markers to authenticate Mora Romagnola meat against meat derived from other 11 pig breeds and wild boars. To our knowledge, the Mora Romagnola Herd Book is one of the first examples that established a direct link between a genetic standard of a breed with the possibility to authenticate mono-breed products using DNA markers with the specific purpose to combat frauds and, indirectly, support the conservation of a livestock genetic resource.

## 1. Introduction

A sustainable strategy for the conservation of animal genetic resources (i.e., autochthonous and usually less efficient breeds compared to cosmopolitan breeds/lines) is based on the marketing of “mono-breed” meat or dairy products, properly labelled for their breed of origin [1,2]. These products are usually sold at a higher price compared to undifferentiated ones, contributing to assuring profitability to the farmers who should be economically interested in raising these less productive animals. The premium price is obtained as the consumers consider positively the link between these breeds and the perceived quality and attributes of their products. On the other hand, this market added value attracts fraudsters that unscrupulously see an economic advantage by selling mis-labelled products to obtain an unjustified additional economic gain. This behaviour is considered one of the most critical problems for a sustainable development of mono-breed production chains, as it produces consumer distrust and undermines the commercial added value of many local and niche animal production chains. Therefore, the development of methods that can authenticate mono-breed products is a key issue for a successful construction of a mono-breed value chain that is able to monitor and defend the integrity of its business model and, in turn, its conservation program [1,3].

Autochthonous breeds are usually small populations due to the low number of females and males in the breeding nuclei and the very low effective population size (*Ne*), mainly due to their genetic histories and subsequent management over the last generations [4,5]. Some breeds might not be completely fixed for typical breed specific features and activities should be oriented to maintain their distinctiveness at the phenotypic and genetic levels [6,7,8].

In Italy, six autochthonous pig breeds (Apulo-Calabrese, Casertana, Cinta Senese, Mora Romagnola, Nero Siciliano and Sarda) are recognised and managed for their conservation by the National Pig Breeders Association (ANAS). Italian local pig breed products are becoming quite important not only for niche markets driven by local consumption and agritourism activities, but also for the interest of large retailers. For example, Cinta Senese meat obtained in 2011 the Protected Denomination of Origin (PDO) label that has contributed to the visibility of its products and to the added value of the meat of this breed, favouring the conservation program of Cinta Senese pigs. On the other hand, this aspect evidenced the need to defend its production chain from fraudsters. For this purpose, a DNA-based method (that uses a polymorphism in a coat colour gene) useful for the authentication of the meat obtained from this breed has been developed and used as a key tool to defend its production chain [9].

**Figure 1:**
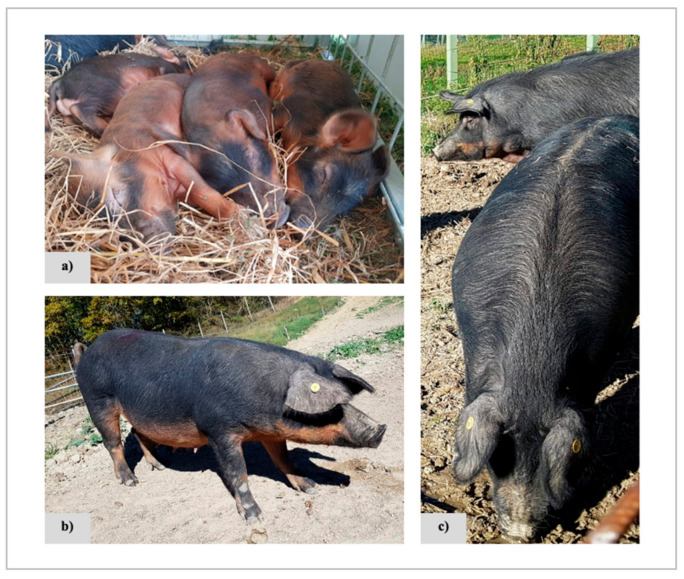
Mora Romagnola pigs: (**a**) piglets with the reddish and black and tan coat colours; (**b**) sow with black coat colour and reddish abdomen (black and tan) and with ears bent forward and parallel to the muzzle; (**c**) a detailed photograph of the bristle back line known as “*Linea Sparta*” (Sparta line).

Mora Romagnola is another autochthonous pig breed, raised mainly in the eastern side of the Emilia-Romagna region (i.e., Romagna), in the north of Italy. Mora Romagnola pigs are recognised as components of the agricultural traditions of this geographical area. At the beginning of the last century, local pig populations in this area (referred to with several local names: e.g., Forlivese, Faentina, Riminese) accounted for more than 300,000 heads. The name ‘’Mora’’, adopted in 1942, refers to the dark (almost black) coat colour (with the dark red–brown colour of the abdomen) that is a characteristic trait of these pigs that could be referred to as black and tan. After the Second World War, only about 20,000 pigs were estimated to belong to this population which was at risk of extinction at the end of the 1980s (due to the substitution of this breed with more productive breeds and lines), when only 18 Mora Romagnola pigs were recovered in one farm. Since then, a preliminary conservation program was established, that, according to historical information, included some unregistered crossbreeding with wild boars and Duroc pigs that contributed to shape, at least in part, the current breed genetic background. Finally, the Herd Book of the Mora Romagnola breed was officially established in 2001 and the breed entered in the conservation program managed by ANAS. Estimated *Ne* of the breed based on molecular markers indicated a very low value, reflecting its genetic history which passed through a strong bottleneck [10,11]. Mora Romagnola pigs are small-medium sized animals, with ears bent forward and parallel to the muzzle, with a dark skin that along the lumbar region hosts black bristles forming a sort of mane (“*Linea Sparta*”) which is the other characteristic trait of the breed (Figure 1). Mono-breed pork products derived from Mora Romagnola pigs are part of an important niche value chain mainly based on a voluntary labelling system that is intrinsically linked to the conservation of this local genetic resource that can only survive due to the added value that its products can obtain at the market.

The black to red coat colours in *Sus scrofa* are mainly determined by alleles at the *Extension* locus, encoded by the melanocortin 1 receptor (*MC1R*) gene [12,13]. The wild type allele (*E^+^*, indicated also as allele 0101; [14]) is the typical form in European wild boars whereas several other alleles are considered the domestic alleles: alleles *E^D1^* (indicated as alleles 0201, 0202 and 0203; [14]) and *E^D2^* (or allele 0301; [14]) that determine the dominant black coat colour (of Asian and European origin, respectively; [12,14,15,16]; allele *E^P^* (identified also as alleles 0501, 0502 and 0503; [13,14]) that is usually reported in spotted and completely white pigs; allele *e* (or allele 0401; [14]), that is the recessive allele associated with the dark-red coat colour of the Duroc breed [12,17]. DNA markers in the *MC1R* gene have been already proposed to authenticate pork from domestic pig breeds or wild boars [12,18,19]. Additional genes affecting coat colour have been described in pigs and some of them might contribute to determine the classical colour observed in the Mora Romagnola pigs [17,20].

Another diagnostic mutation that could distinguish domestic pigs from wild boars is in the nuclear receptor subfamily 6 group A member 1 (*NR6A1*) gene. A missense mutation (g.299084751C>T), that causes the p.P192L amino acid substitution, fixed in commercial breeds for the domestic allele, is associated with an increased number of vertebrae compared to pure wild boars (21–23 versus 19 vertebrae; [21]). Wild boars are fixed or almost fixed for the wild type allele [16,19,22].

Applied research activities focused on the sustainable conservation of animal genetic resources have been recently focused on the characterisation of European autochthonous pig breeds including phenotypic and genotypic descriptions of local populations, starting from small groups of pigs that might be representative of the whole breed populations [9,10,15,20,23,24]. As far as we know, however, no specific attempts have been addressed to obtain a complete characterisation of a whole pig breed, in order to establish an efficient and direct monitoring approach that might be able to better implement conservation activities.

In this study we (i) monitored phenotypically the Mora Romagnola population to evaluate the compliance to the breed standard, (ii) characterised this breed population for polymorphisms in the *MC1R* and *NR6A1* genes by genotyping almost all living sows and boars registered to the Herd Book, (iii) included information of these DNA markers in the Mora Romagnala breed Herd Book and (iv) described how these markers can be linked to the genetic authentication system that was designed to combat fraud that undermines the Mora Romagnola mono-breed production system. These activities were specifically carried out to lead this local pig breed towards a sustainable conservation of its genetic uniqueness by involving direct actions that included almost the whole breeding population.

## 2. Materials and Methods

### 2.1. Mora Romagnola Population over the Years

Information on the number of pigs of the Mora Romagnola breed registered to the Herd Book from 2001 (the year of official constitution of the breed) to 2019 was retrieved from ANAS records (Table S1), available in the Mora Romagnola Herd Book database [25]. Animals were classified into breeding pigs (boars and sows) and young pigs, that, according to the breed Herd Book definition, are males that were of less than 8 months of age or females until the first farrow (their final destination was not established at the date of the recording: they could become breeding animals, i.e., boars or sows, or they could be slaughtered). The final recording date for statistics and destination of the animals was after the end of each corresponding year, when the collection of the information from the registered farms was completed. Average age of the registered boars and sows was calculated from the records available in the Herd Book database.

### 2.2. Phenotyped Animals

Phenotyping of the pigs was carried out in the years 2017–2019. Complete photographic records and/or detailed morphological descriptions based on three breed specific traits [i.e.,: coat colour, “*Linea sparta*” and ear shape/position (Figure 1)] were obtained in this study for 826 Mora Romagnola pigs raised in 27 farms (Table S2), located in different administrative geographic units [provinces (Figure S1)]. Phenotyping classes were defined according to the presence or absence of the breed specific standards of the three traits: (i) for the coat colour in adult (black and tan) or in young pigs (black and tan or self-red, that changes in adults to become black and tan); (ii) for the position of the ears in both adult or young pigs (ears bent forward and parallel to the muzzle as standard trait or carried in other positions, not considered a part of the morphological standard); (iii) for the “*Linea sparta*” in both adult and young pigs. The Herd Book of the breed reports these three traits as descriptors of breed specific features. The adult breeding pigs of this group (357 pigs: 110 boars and 247 sows) were also genotyped, as described below. The young pigs (n. = 469) were only phenotyped.

### 2.3. Genotyping of MC1R and NR6A1 Gene Markers

The genotyped population included 110 boars and 247 sows registered in the Herd Book as components of the breeding nucleus. These pigs were also among the group of the 826 phenotyped Mora Romagnola pigs. Considering that generations overlapped over the years, the genotyped boars and sows constituted about 98% of all active and living males and 92% of all active females of the breeding nucleus of the whole breed.

To compare genotyping data (see below) between the Mora Romagnola breed and other breeds and populations, this study included a total of other 861 animals belonging to cosmopolitan pig breeds or populations (n. 49 Italian Large White; n. 44 Italian Landrace; n. 30 Italian Duroc n. 26 Pietrain; n. 31 Belgian Landrace; n. 18 Hampshire), other Italian local pig breeds (n. 101 Apulo-Calabrese; n. 161 Casertana; n. 122 Cinta Senese; n. 108 Nero Siciliano; n. 58 Sarda) and Italian wild boars (n. 113). Genotyping information of these animals were already included in other studies [15,16,19,26]. In addition, genotyping results from another population of 74 Mora Romagnola pigs, sampled in the years 2010–2014 and described by Ribani et al. (2019) were compared to those obtained in this study from the same breed.

Hair roots were collected from all Mora Romagnola pigs. No ethical permit was needed for this study as biological specimens were collected as part of the routine work of the Mora Romagnola Herd Book. DNA extraction was carried out using the Wizard Genomic DNA Purification kit (Promega Corporation, Madison, WI, USA) or using a standard phenol–chloroform method.

Five autosomal polymorphisms were genotyped: three single nucleotide polymorphisms (SNPs) and one insertion/deletion (indel) in the *MC1R* gene that, on the whole, can distinguish all major alleles at the *Extension* locus (*E^+^*, *E^D1^*, *E^D2^*, *E^P^* and *e*) described by [12,13]; one missense mutation in the *NR6A1* gene (g.299084751 C>T or p.P192L) which is the causative mutation of the QTL for number of vertebrae, identified on porcine chromosome 1 [21]. PCR conditions and primers were already reported [16,18,26]. Three fragments were amplified for the *MC1R* gene. One amplicon was 196 bp long and included SNP c.367G>A which differentiates the wild allele *E^+^* from alleles *E^D2^* and *E^P^*. This SNP was analysed by PCR-Restriction Fragment Length Polymorphism (RFLP) using restriction enzyme *Bsp*HI (recognised sequence: TCATGA) which cuts *E^D2^* and *E^P^* but not *E^+^*. The second *MC1R* amplicon (154 bp long) included two SNPs (c.727G>A and c.729G>A) that were analysed by PCR-RFLP. The first SNP (c.727G>A), that differentiates allele *e* from all other alleles, was genotyped digesting the amplified fragment with restriction enzymes *Hha*I (recognised sequence: GCGC) that cuts all alleles except allele *e*. The second SNP (c.729G>A) that distinguishes all alleles from allele *e* and *E^D1^* was analysed by digesting the obtained amplicon with restriction enzyme *Bst*UI (recognised sequence: CGCG) that cuts all alleles except alleles *e* and *E^D1^*. The third *MC1R* amplicon included the indel that discriminates allele *E^P^* from allele *E^D2^*. The amplified DNA was analysed by fragment length analysis on a capillary sequencer (ABI PRISM 3100 Avant Genetic Analyzer, Applied Biosystems): allele *E^D2^* was of 168 bp whereas allele *E^P^* was of 170 bp due to an insertion of CC at position 67 of the coding region. The *NR6A1* polymorphism (g.299084751C>T) was genotyped by PCR-RFLP from a PCR product of 203 bp using restriction enzyme *Msp*I that cuts the amplified fragment when the wild type allele is present. To confirm the genotyping results, 3-4 fragments, obtained from each primer pairs and showing different genotypes at the PCR-RFLP or fragment analyses, were sequenced using a Sanger sequencing approach, as previously described [19,26].

### 2.4. Data Analyses

Allele and genotype frequencies were calculated for each breed/population at the two investigated loci. The Hardy–Weinberg equilibrium was evaluated with the HWE software program (Linkage Utility Programs, Rockefeller University, New York, NY, USA).

Multidimensional scaling (MDS) was used to evaluate the relationships among the analysed breeds. Briefly, MDS was carried out in R v.3.4.4 previa computation of a dissimilarity matrix D (in which each value represents the Euclidean distance *d* between two populations) based on allele frequencies. The first two MDS components (C1 and C2) were evaluated.

GENEPOP software version 4.0.7 [27] was used to calculate population pairwise *F_st_* genetic distance and G genic differentiation for each population pair (exact G test) that is a modification of the Fisher’s exact probability test [27]. Markov chain parameters used were: Dememorisation: 10,000; Batches: 100; Iterations per batch: 5000) including the two gene markers.

Probability to incorrectly assign an unknown meat sample to populations different from Mora Romagnola or crossbred products of this breed versus all other populations (error rate: ER) was calculated using the following formulas [9], reported for both loci:

ER(MC1R) = 1 − |δ*_MC1R_*| (1)

where |δ*_MC1R_*| is the absolute allele frequency difference between the sum of allele frequency of the two Mora Romagnola *MC1R* alleles (*E^+^* and *e*) observed in the other breeds with respect to what is observed in Mora Romagnola breed, after the culling of a few animals carrying other alleles (i.e.,: f(*E^+^*) + f(*e*) = 1);

ER(NR6A1) = 1 − |δ*_NR6A1_*| (2)

where |δ*_NR6A1_*| is the absolute allele frequency difference at the *NR6A1* gene between Mora Romagnola and the other breeds;

ERc = ER(MC1R) × ER(NR6A1)(3)

is the combined error rate derived by the two genes.

Vice versa, the probability to correctly assign an unknown meat sample to Mora Romagnola (PMR) was calculated using the following formulas, defined following [9,19]:

PMR(MC1R) = 1 − [f(*E^+^*/*E^+^*) + f(*E^+^*/*e*) + f(*e*/*e*)] (4)

where f(*E^+^*/*E^+^*), f(*E^+^*/*e*), f(*e*/*e*) are the frequency of occurrence of pigs with *MC1R* genotype *E^+^*/*E^+^*, *E^+^*/*e* or *e*/*e* in the other populations;

PMR(NR6A1) = 1 − [f(*T/T*)] (5)

where f(*T/T*) is the frequency of pigs with *NR6A1* genotype *T/T* in the other populations.

## 3. Results

### 3.1. Phenotypic Characterisation of the Mora Romagnola Population

**Figure 2:**
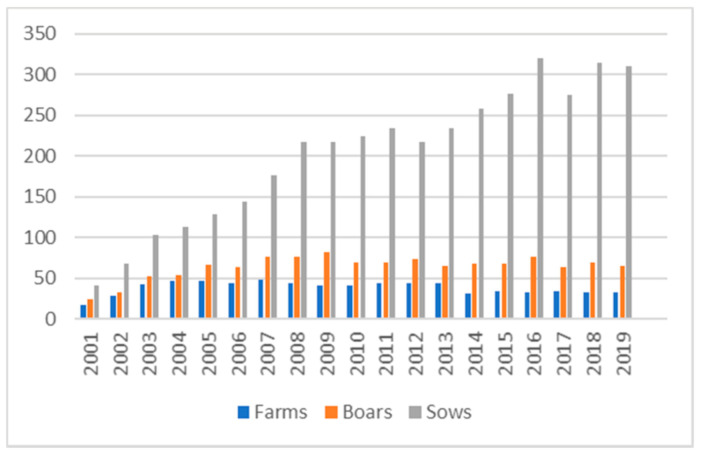
Number of Mora Romagnola Herd Book registered farms, boars and sows (y axis) from 2001 to 2019 (x axis). Detailed information is reported in Table S1.

Figure 2 summarises the number of farms rearing Mora Romagnola pigs and the number of boars and sows registered to the breed Herd Book, by years, since its constitution. Table S1 reports these numbers as well as the number of young pigs of the breed. The total number of registered reproducers has been increasing since the constitution of the breed Herd Book in 2001, mainly due to an increase in the number of registered sows and young pigs. Therefore, a recurrent phenotyping monitoring is needed for the maintenance of the breed standard and the distinctive characteristics of the Mora Romagnola pig population.

The average age of the sows that were registered in the years 2017–2019 (the years of the phenotyping activities) and that delivered in this period was 2.3 years, whereas the average age of the boars in the same period was 2.2 years. Thus, Mora Romagnola can be considered a small population where generations overlap.

The phenotypic characterisation that occurred over the same period was based on a total of 826 pigs (357 adult breeding animals and 469 young pigs; Table S3). All adult pigs (male and female breeding pigs) had the standard coat colour except one sow (0.3% of the adult pigs) that had red coat colour. Despite the non-standard coat colour, this animal was still listed in the Herd Book due to a delay in the update of the information available in the database, considering that young pigs can be registered even if they have this colour that, however, is not tolerated in the breeding animals. Red coat colour was recorded in about 8.9% of the young pigs. For the other two traits (ears position and presence/absence of “*Linea sparta*”), all adult pigs had the standard phenotype, whereas phenotypic variability was observed in the young pigs. About 11% of these animals had half-hanging or raised ears and about 9% of this group did not have “*Linea sparta*”.

### 3.2. MC1R and NR6A1 Allele and Genotype Frequencies in Mora Romagnola and Breed Standard Genotypes

The observed genotypes at the *MC1R* and *NR6A1* genes and their frequencies in the genotyped Mora Romagnola breeding pigs are reported in Table 1. The two genes were not completely fixed for one allele. Two major alleles were identified at the *MC1R* gene: *e* (frequency of 0.790) and *E^+^* (frequency of 0.192). Therefore, alleles *e* and *E^+^* could be considered the breed specific alleles for the following reasons: (1) the genetic history of the Mora Romagnola breed that experienced, at the beginning of its recovery, crossbreeding with Duroc pigs and wild boars, from which these two alleles may have been introgressed; (2) the observed high frequency of alleles *e* and *E^+^* in the Mora Romagnola population. Another two alleles were observed at this gene but with very low frequency (*E^D2^* = 0.017; *E^D1^* = 0.001). Pigs carrying the dominant *E^D1^* and *E^D2^* alleles appeared a little bit darker than the animals carrying the other *MC1R* alleles, but they could not be clearly phenotypically distinguished from all other pigs of this breed.

Only two pigs carried the wild type allele at the *NR6A1* polymorphic site (one in homozygous and one in heterozygous state). The frequency of the wild type allele (allele g.299084751C) was therefore very low (0.004). Thus, the breed characteristic allele could be considered the domestic allele (g.299084751T), as already reported in many other domestic breeds [16,19].

The pigs that carried the breed non-specific alleles at the *MC1R* gene were 11 sows and one boar, raised in six different farms: three farms had more than one of these pigs: one had four sows, one had three sows and another one had two sows listed in this group. The boar with genotype *E^D2^*/*e* was raised in a small farm in the province of Ravenna where all other sows carried alleles *E^+^* and/or *e*. *NR6A1* allele C was carried by two sows raised in two different farms. All these animals were excluded from the breed Herd Book and were subsequently slaughtered.

After this genotyping activity and subsequent actions, the breed could be virtually considered free from other alleles that are not the breed-specific alleles (i.e., *e* and *E^+^* for the *MC1R* gene and T for the *NR6A1* gene). Based on these results, the standard of the Mora Romagnola Herd Book was modified including within the description of the breed, the allowed genotypes at the *MC1R* gene (*e*/*e*, *E^+^*/*e* and *E^+^*/*E^+^*) and at the *NR6A1* gene (T/T), in addition to the other breed-specific phenotypic descriptors.

### 3.3. MC1R and NR6A1 Allele Frequencies in Mora Romagnola and Other Pig Breeds and in Wild Boars

**Figure 3:**
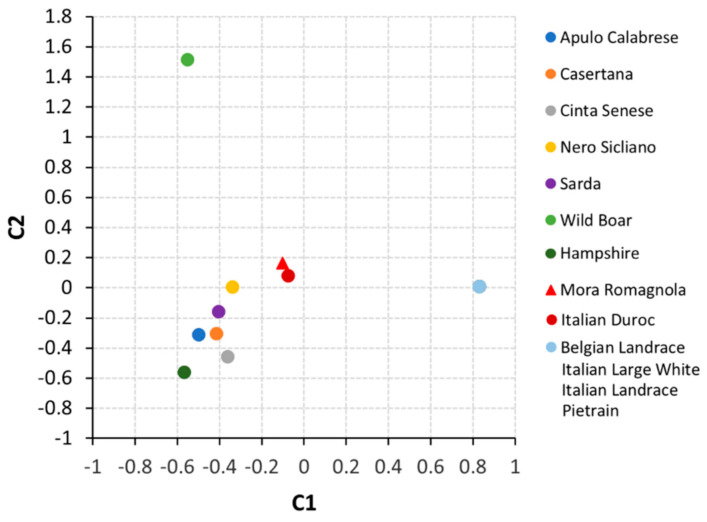
Multidimensional scaling (MDS) plot obtained using allele frequencies at the *MC1R* and *NR6A1* genes in Mora Romagnola and other 11 pig breeds and wild boars. Components 1 and 2 (C1 and C2) are represented. The cosmopolitan breeds with the same allele frequencies at the two genes (Belgian Landrace, Italian Large White, Italian Landrace and Pietrain) are grouped in just one point.

Genotyping data at the *MC1R* gene in the Mora Romagnola breed respected the Hardy‒Weinberg equilibrium (*p* > 0.10). A comparison of the allele frequencies at the *MC1R* and *NR6A1* genes between Mora Romagnola and several other pig breeds and wild boars is summarised in Table 2. Mora Romagnola allele frequencies have been re-calculated on a total of 342 breeding pigs that were maintained in the Herd Book after the phenotyping and genotyping analyses that excluded 15 animals (one after the phenotyping and 14 after the genotyping activities). Genotyping data from all other breeds (six cosmopolitan breeds and five Italian local breeds) and from an Italian wild boar population sampled in the Emilia-Romagna region have been compiled from our previous works [15,16,19,26]. Figure 3 shows the MDS plot derived from the allele frequencies at these two genes in Mora Romagnola and 11 other pig breeds and in wild boars.

Mora Romagnola had |δ*_MC1R_*| > 0.80 with four out of five local breeds and five out of six cosmopolitan breeds. Sarda had |δ*_MC1R_*| = 0.638 and Italian Duroc, that is fixed for allele *e*, had |δ*_MC1R_*| = 0.191. Wild boars had both *E^+^* and *e* alleles but with opposite extreme frequencies than those observed in Mora Romagnola, i.e., allele *E^+^* was the most frequent allele, 0.925, whereas allele *e* was the less frequent variant observed in this population). The highest |δ*_NR6A1_*| was observed against the wild boar population, that was almost fixed for the wild type allele (allele C). This allele was also observed in some of the local breeds. All cosmopolitan breeds were fixed for allele T, that is the same allele fixed in Mora Romagnola.

Allele frequencies in the 2010–2014 Mora Romagnola population, derived by previous studies, matched the results reported in the current larger investigation. Two *MC1R* alleles were identified: alleles *E^+^* and *e* had frequencies of 0.176 and 0.824 (versus 0.192 and 0.809 in the 2017–2019 sample). Only allele T was detected at the *NR6A1* gene [16].

Pairwise *F_st_* measures indicated that all comparisons of Mora Romagnola breed against all other breeds and populations were significant (*p* < 0.001) (Table 3). *MC1R* was informative in all comparisons, whereas *NR6A1*, that was fixed or almost fixed for the T allele in most breeds, had a limited informativeness. The *F_st_* value was quite low against the Italian Duroc breed that had also the lowest |δ*_MC1R_*| value. Genic differentiation for each population pair (exact G test) was highly significant in all pairwise analyses except against the 2010–2014 Mora Romagnola dataset, as also confirmed by the negative and close to zero *F_st_* value, further indicating that there were no differences between the two Mora Romagnola populations sampled in different time windows.

### 3.4. Usefulness of MC1R and NR6A1 to Differentiate Mora Romagnola Meat

Based on the genotyping data obtained in the different pig populations, we evaluated if *MC1R* and *NR6A1* gene markers could be useful to differentiate Mora Romagnola meat from meat of other pig breeds and from wild boars (Table 4). In this first analysis, we have not considered the Italian Duroc breed as the *F_st_* estimate indicated that it was the closest breed to Mora Romagnola.

*MC1R* was very informative against all breeds. The unique allele frequency distribution at this locus in the Mora Romagnola breed made it possible to estimate a very low error rate defined as the probability to incorrectly assign an unknown meat sample to populations different from Mora Romagnola. The error rate was reduced when combined with that derived from *NR6A1*, particularly against wild boars that, on the contrary, had a lower effectiveness from the *MC1R* gene due to the presence in high frequency of the *E^+^* allele. Sarda breed gave the combined highest error rate, due to the heterogeneity at the two analysed loci and high frequencies of both *E^+^* and *e*, compared to all other breeds (Table 4).

On the other hand, the probability to correctly assign an unknown meat sample to Mora Romagnola breed considering one or the other locus was equal to one against eight breeds/populations (Casertana, Cinta Senese, wild boars, Italian Large White, Italian Landrace, Pietrain, Belgian Landrace and Hampshire) and >0.90 against Apulo Calabrese, Nero Siciliano and Sarda (Table 4).

Italian Duroc was the breed that determined the highest error rate (0.809). Based on the *MC1R* and *NR6A1* loci, meat derived from Italian Duroc or Mora Romagnola could not be distinguished.

## 4. Discussion

The Mora Romagnola pig breed is nowadays considered an icon of the traditional agricultural sector of the Emilia-Romagna region. Its products fill an important local niche of the market where quality and tradition are considered added values that make possible the sustainable conservation of this local breed. However, this value chain has been affected by several instances of fraud due to mis-labelling of products not derived from Mora Romagnola pigs. This type of fraud is considered one of the main problems for the economic maintenance of Mora Romagnola farms and processors.

Since the constitution of the breed Herd Book, the Mora Romagnola population has been increasing in terms of number of heads even if the total number of animals is still quite limited (Figure 2). The relatively low number of heads, the increasing market demand of Mora Romagnola products and the low performances of the Mora Romagnola pigs in terms of growth rate, feed and reproduction efficiencies have also driven some farmers to the use of better performing animals in crossbreeding plans, as also happened in several similar situations in different local breeds (e.g., [28]).

Based on these critical conditions, in order to preserve the phenotypic and genetic uniqueness of the breed, monitoring and genotyping programs have been started with the final aim to provide information and tools to support the Mora Romagnola value chain, starting from the breeding nucleus of its population. Within this program, almost all Mora Romagnola farms were inspected, and animals were phenotyped to preliminarily evaluate the level of potential admixture (even based on morphological analyses and on only two DNA markers) from other breeding stocks. This activity did not identify any specific problems or drifts towards incomplete morphological correspondence to the Herd Book standard. The breeding nucleus (boars and sows) was clearly in compliance with the Herd Book standard even if the routine phenotyping system of morphological traits of the breed does not record all the details that we included in this study. That means that the few out-of-type animals, that could eventually appear sometimes, are then efficiently identified, culled and not admitted to the breeding nucleus. Only one sow still listed among the breeding animals had the red coat colour that is not allowed by the breed standard of adult pigs. Young pigs can have red/reddish colour that, after a few months, usually changes to the classical black and tan standard colour (Figure 1). The mechanisms by which only some of the young pigs can have red colour, that in most cases changes to the standard colour after a few months, are not known and need to be investigated. The *agouti* locus might play a role in determining the standard colour of the adult animals and, potentially, allele(s) inherited from the wild boars could contribute to the peculiar coat colour phenotype of this breed. However, whole genome resequencing data obtained for this breed [20,24] did not evidence any clear signature of selection patterns in the correspondence of the agouti signalling protein (*ASIP*) gene that is the genetic determinant of the *agouti* locus. Other genes could contribute to determine the Mora Romagnola characteristic coat colour phenotype. Another phenotypic trait, used to identify the animals of this breed, is the so called “*Linea sparta*”, that is a sort of mane of hairs on the back of the pigs. The genetic determinant(s) of this specific trait is (are) not known yet and other studies are needed to clarify this peculiar phenotype. Another trait that is considered to be breed-specific is the position of the ears. Young Mora Romagnola pigs could have more frequently half hanging ears that, when the animals grow up, could change position towards the standard phenotype.

The results obtained by the genotyping of markers in the *MC1R* and *NR6A1* genes confirm, to some extent, the genetic isolation of the breed. Two *MC1R* alleles can be considered the breed specific alleles: *E^+^*, that is also common in wild boars [12,16,19] and *e*, that might contribute to the reddish phenotype (in part evident in the young and adult Mora Romagnola animals) and that is fixed in Duroc pigs [12,19,26]. The high frequency of both alleles in Mora Romagnola breed confirms its genetic history where wild boars and Duroc blood were probably introduced in the early breed population. The genetic closeness between Mora Romagnola and Italian Duroc breeds has been already reported by whole genome sequencing results [20,24]. Allele frequency observed in the animals sampled in 2017–2019 did not differ from the allele frequencies at the same two loci obtained in past generations, indicating that the population is quite stable and there are no specific pressures against one or the other allele at the *MC1R* gene. It is however not completely clear what could be the precise role of these alleles in determining the coat colour of the Mora Romagnola animals. Animals that have the three different genotypes at this locus (*e*/*e*, *E^+^*/e and *E^+^*/*E^+^*) do not have a clear distinct coat colour in adult pigs. It could be possible that different *MC1R* genotypes are associated with some pigmentation differences that are observed in young pigs. Further studies should clarify this question.

Genotyping of the *MC1R* and *NR6A1* markers identified only few animals (14 out of 357 breeding pigs) that did not carry alleles that were expected in the breed and that were considered breed-specific alleles. Two other *MC1R* alleles have been detected: *E^D2^* and *E^D1^*, with a very low frequency (Table 1). Their effect on coat colour might be masked by other genes that determine or that contribute to the standard coat colour of the adult animals. These alleles could be derived by the use of some other black pigs in few crossbreeding cases that, according to this genetic evidence, might have been quite infrequent. Two other pigs carried the wild type allele at the *NR6A1* gene. This allele is considered the original form in wild boars. It was surprising to note that, despite the fact that the wild type allele at the *MC1R* gene had a relatively high frequency, the same was not the case for the wild type allele at the other locus. As the *NR6A1* T allele (the domestic allele) is associated with an increased number of vertebrae (and with an increased number of teats; [21]), it could be possible that selection towards domesticated traits, related to improved performances, could have contributed to indirectly eliminate the wild-type form from the breed.

Based on the results obtained in this study, ANAS redefined the Herd Book standard of the breed including, as additional descriptors, the genotypes at the *MC1R* and *NR6A1* genes: pigs belong to the Mora Romagnola breed if, in addition to the three main breed-specific morphological traits (black and tan coat colour of the adult pigs, hanging ears and “*Linea sparta*”), they have only the *MC1R E^+^*/*E^+^*, *E^+^*/*e* or *e*/*e* genotype and the *NR6A1* T/T genotype. The fixation of these two loci did not create any problems in the reduction in genetic variability of the breed as just 3.9% of the breeding animals were excluded. The “genetic cost”, however, has been compensated by the fact that, starting from the clear definition of the Mora Romagnola breeding nucleus, it was possible to establish a genetic link useful for the authentication of the Mora Romagnola products. A DNA test can be easily implemented to detect the *MC1R* and *NR6A1* genotypes. Only meat products that can have the allowed genotypes at these two loci can be considered to be obtained from Mora Romagnola pigs. A similar approach has been already described in another Italian local pig breed, Cinta Senese (mainly raised in Tuscany), which was characterised for another DNA markers associated with its belted phenotype [9]. The Herd Book of Cinta Senese breed has been modified following the same strategy described in the Mora Romagnola breed, even if the larger size of the Tuscany breed could make it possible to genotype only a smaller fraction of the breeding population [9].

DNA-based authentication systems of mono-breed products are mainly designed on the distribution and frequency of informative alleles and genotypes in the different animal populations that need to be distinguished and separated by the targeted breed. The level of fixation of specific markers in the targeted population is also very relevant for the practical and successful implementation of the system. A few examples that used coat colour gene markers have been already reported in other livestock species, e.g., cattle and sheep [29,30]. One of the advantages of these systems is that they can be implemented in practice and that they can discourage fraud, considering that fraudsters, at these levels, cannot usually compete against DNA authentication methods [1].

The method designed for the Mora Romagnola breed based on *MC1R* and *NR6A1* relies on the related allele frequency distribution in many pig breeds and populations. Even if, in theory, the error rate that could be expected from the combined *MC1R* and *NR6A1* genotyping approach is not zero and the probability to correctly assign an unknown meat sample to Mora Romagnola is not 100%, in practice the system can be useful to identify the major potential fraud due to the mis-labelling of non-Mora Romagnola products. It is also clear that potential fraud would not be originated by the substitution of meat coming from other local breeds (that, according to their genetic structure could give a quite high error rate) but only using cheaper meat originating from commercial populations or cosmopolitan breeds that carry the *E^P^* or *E^D2^* alleles at the *MC1R* gene, that are not present or allowed in the Mora Romagnola breed. One problem could be due to the Duroc breed whose products cannot be distinguished from those of Mora Romagnola origin using this genotyping system. It is however well known that Duroc pigs are only used as breeding animals (mainly as sires) in commercial crossbreeding plans and the number of pigs of this breed that are slaughtered and commercialised is very limited. Hybrid commercial pigs could eventually carry only one copy of allele *e* if the animal is originated by crossing a Duroc with another commercial line, which might be derived from cosmopolitan breeds that carry *E^P^* or *E^D2^*.

This system, currently based on just two genes, could be further refined adding other markers if their discrimination potential to distinguish Mora Romagnola pigs, and in turn Mora Romagnola derived products, from all other breeds or commercial lines is demonstrated. For example, if polymorphisms associated with “*Linea Sparta*” would be identified, these markers could be the right candidates to be included in this system as they might also strengthen the phenotypic uniqueness of this breed. DNA markers in SNP chip panels could also provide information useful for the authenticity of mono-breed products (e.g., [31,32]), including Mora Romagnola products, but the use of their genotyping information to discriminate the pigs that could be registered to the Herd Book would not be practical.

## 5. Conclusions

To our knowledge this is one of the first study that involved almost the complete breeding population of a breed with the final aim to develop a system able to link the Herd Book and the authentication of mono-breed products. Two molecular markers had useful features for this purpose in the Mora Romagnola pig breed and were used as breed-specific descriptors. The final aim was to combat fraud in the meat-derived niche value chain. The results of genotyping activities showed that it was possible to fix *MC1R* and *NR6A1* alleles in this breed population without affecting its genetic variability too much. The activities that have been carried out in Mora Romagnola can represent an important case study for similar approaches in other small breeds, which should be adapted according to the genetic structure and potential sources of other genetic materials used by fraudsters. The monitoring of the meat products labelled as being derived from Mora Romagnola pigs and sold in niche markets is currently under way.

## Figures and Tables

**Table 1:** Genotypes at the *MC1R* and *NR6A1* genes observed in the analysed Mora Romagnola pigs.

***MC1R* Genotypes**	**No. of Pigs**	**Genotype Frequency**
*e/e*	225 ^1^	0.630
*E^+^/e*	109	0.305
*E^+^/E^+^*	11	0.031
*E^+^/E^D2^*	5	0.014
*E^D2^/e*	5	0.014
*E^+^/E^D1^*	1	0.003
*E^D1^/E^D2^*	1	0.003
***NR6A1* Genotypes**	**No. of Pigs**	**Genotype Frequency**
T/T	355 ^1^	0.994
T/C	1	0.003
C/C	1	0.003

^1^ The sow that had red coat colour that was described in the previous paragraph (phenotyping characterisation) had genotype *e*/*e* and T/T at the *MC1R* and *NR6A1* genes, respectively.

**Table 2:** Allele frequencies at the *MC1R* and *NR6A1* genes in different breeds and populations.

Breeds/Populations	No. of Pigs	*MC1R* Alleles						*NR6A1* Alleles		
		*E^+^*	*e*	*E^D1^*	*E^D2^*	*E^P^*	|δ*_MC1R_*| ^1^	T	C	|δ*_NR6A1_*| ^1^
Mora Romagnola	342 ^2^	0.192	0.809	0.000	0.000	0.000	-	1.000	0.000	-
Apulo Calabrese	101	0.040	0.035	0.000	0.856	0.069	0.925	0.856	0.144	0.144
Casertana	161	0.124	0.019	0.000	0.767	0.090	0.857	0.938	0.062	0.062
Cinta Senese	122	0.004	0.041	0.004	0.820	0.131	0.955	1.000	0.000	0.000
Nero Sicliano	108	0.120	0.005	0.056	0.630	0.190	0.875	0.718	0.282	0.282
Sarda	58	0.302	0.060	0.060	0.578	0.000	0.638	0.991	0.009	0.009
Wild Boar	113	0.925	0.022	0.000	0.004	0.049	0.733–0.787 (0.053)	0.018	0.982	0.982
Italian Large White	49	0.000	0.000	0.000	0.000	1.000	1.000	1.000	0.000	0.000
Italian Landrace	44	0.000	0.000	0.000	0.000	1.000	1.000	1.000	0.000	0.000
Italian Duroc	30	0.000	1.000	0.000	0.000	0.000	0.191	1.000	0.000	0.000
Pietrain	26	0.000	0.000	0.000	0.000	1.000	1.000	1.000	0.000	0.000
Belgian Landrace	31	0.000	0.000	0.000	0.000	1.000	1.000	1.000	0.000	0.000
Hampshire	18	0.000	0.000	0.000	1.000	0.000	1.000	1.000	0.000	0.000

^1^ Absolute delta |δ| allele frequency differential of the alleles between Mora Romagnola breed (sampled in 2017–2019) and all other breeds and populations. This parameter was calculated for the two loci separately: |δ*_MC1R_*| and |δ*_NR6A1_*|. |δ*_MC1R_*|: for *MC1R*, two alleles were considered to be representative for the Mora Romagnola breed; therefore, the comparison was made combining the allele frequency of *E^+^* and *e* in the other breeds. In the wild boar comparison, |δ*_MC1R_*| was obtained calculating the allele frequency differential for the two alleles separately. In parenthesis, |δ*_MC1R_*| in the combined analysis is also reported. ^2^ Number of breeding pigs that were maintained in the Herd Book after the phenotyping and genotyping analyses.

**Table 3:** Pairwise *F_st_* and genic differentiation (exact G test) comparing Mora Romagnola data derived by the 2017-2019 sampling versus all other investigated breeds and populations.

Breeds/Populations	*F_st_* ^2^	*p* of the G Test
Mora Romagnola (2010–2014) ^1^	−0.003 ^3^	0.621
Apulo Calabrese	0.663 (0.694; 0.267)	<0.0001
Casertana	0.623 (0.646; 0.090)	<0.0001
Cinta Senese	0.679	<0.0001
Nero Sicliano	0.572 (0.596; 0.449)	<0.0001
Sarda	0.564 (0.565; 0.024)	<0.0001
Wild Boar	0.848 (0.683; 0.991)	<0.0001
Italian Large White	0.756	<0.0001
Italian Landrace	0.754	<0.0001
Italian Duroc	0.107	<0.0001
Pietrain	0.745	<0.0001
Belgian Landrace	0.747	<0.0001
Hampshire	0.740	<0.0001

^1^ Pairwise analyses between the Mora Romagnola genotyping data obtained from the 2017–2019 samples were also carried out against the Mora Romagnola data obtained from the 2010–2014 population. ^2^ *F_st_* values were estimated including the two genes: the estimates obtained with the *MC1R* and *NR6A1* genes are reported in brackets when both loci were informative. When only *MC1R* was informative, only one value was reported. ^3^ *F_st_* comparison was not significant (the negative value does not have any biological meaning and should be considered as equal to zero). In all other pairwise analyses, the test was significant (*p* < 0.001).

**Table 4:** Probability to incorrectly assign an unknown meat sample to populations different from Mora Romagnola (error rate) and probability to correctly assign an unknown meat sample to Mora Romagnola in comparison to all other breeds and population investigated as determined by genotyping *MC1R* and *NR6A1* gene markers.

Breeds	ER(MC1R) ^1^	ER(NR6A1) ^2^	ERc ^3^	PMR(MC1R) ^4^	PMR(NR6A1) ^5^
Apulo Calabrese	0.075	0.856	0.064	0.990	0.277
Casertana	0.143	0.938	0.134	1.000	0.081
Cinta Senese	0.045	1.000	0.045	1.000	0.000
Nero Sicliano	0.125	0.718	0.090	0.963	0.435
Sarda	0.362	0.991	0.359	0.948	0.017
Wild Boar	0.240 (0.947)	0.018	0.004 (0.017)	0.106	1.000
Italian Large White	0.000	1.000	0.000	1.000	0.000
Italian Landrace	0.000	1.000	0.000	1.000	0.000
Italian Duroc	0.809	1.000	0.809	0.000	0.000
Pietrain	0.000	1.000	0.000	1.000	0.000
Belgian Landrace	0.000	1.000	0.000	1.000	0.000
Hampshire	0.000	1.000	0.000	1.000	0.000

^1^ Error rate calculated from the *MC1R* genotyping data. In the comparison with wild boar data, |δ| was obtained from the alleles *E^+^* and *e* was averaged (Table 2). The result obtained considering the combined |δ| derived by summing *E^+^* and *e* allele frequencies in wild boars is reported in parenthesis. ^2^ Error rate calculated from the *NR6A1* genotyping data. ^3^ Combined error rate from the two loci. ^4^ The probability to correctly assign an unknown meat sample to Mora Romagnola based on the *MC1R* genotyping data. ^5^ The probability to correctly assign an unknown meat sample to Mora Romagnola based on the *NR6A1* genotyping data.

## Data Availability

Data is contained within the article or supplementary material.

## Supplementary Materials

The following are available online at https://www.mdpi.com/2076-2615/11/2/526/s1, Figure S1: Geographic distribution of Mora Romagnola farms in the different administrative units (Provinces) of the north of Italy, Emilia Romagna Region: red dots indicate the geographic localisation of the farms in which pigs were phenotyped and sampled in the years 2017–2019. Table S1: Number of Mora Romagnola Herd Book registered farms, boars, sows and young pigs per year (from 2001 to 2019; [25]. Table S2: Number of Mora Romagnola pigs that were phenotyped in the years 2017–2019, distributed in different farms and provinces. The breeding animals were also genotyped. Table S3: Number of Mora Romagnola pigs with different phenotypes for three recorded exterior traits (coat colour, ear position and “*Linea sparta*”).
