# Supplementary material for: Redefinition of the Mora Romagnola Pig Breed Herd Book Standard Based on DNA Markers Useful to Authenticate Its “Mono-Breed” Products: An Example of Sustainable Conservation of a Livestock Genetic Resource
Source: Animals (Basel). 2021 Feb 18;11(2):526. doi: 10.3390/ani11020526 (PMC7923016; doi:10.3390/ani11020526)
Supplement: Supplementary file 1 [file animals-11-00526-s001.pdf]

animals

# Supplementary Material

# Redefinition of the Mora Romagnola Pig Breed Herd Book Standard Based on DNA Markers Useful to Authenticate Its "Mono-Breed" Products: An Example of Sustainable Conservation of a Livestock Genetic Resource

**Silvia Tinarelli, Anisa Ribani, Valerio Joe Utzeri, Valeria Taurisano, Claudio Bovo, Stefania Dall'Olio, Francesco Nen, Maurizio Gallo, and Luca Fontanesi**

**Figure S1.** Geographic distribution of Mora Romagnola farms in the different administrative units (Provinces) of the North of Italy, Emilia Romagna Region: red dots indicate the geographic localization of the farms in which pigs were phenotyped and sampled in the years 2017-2019.

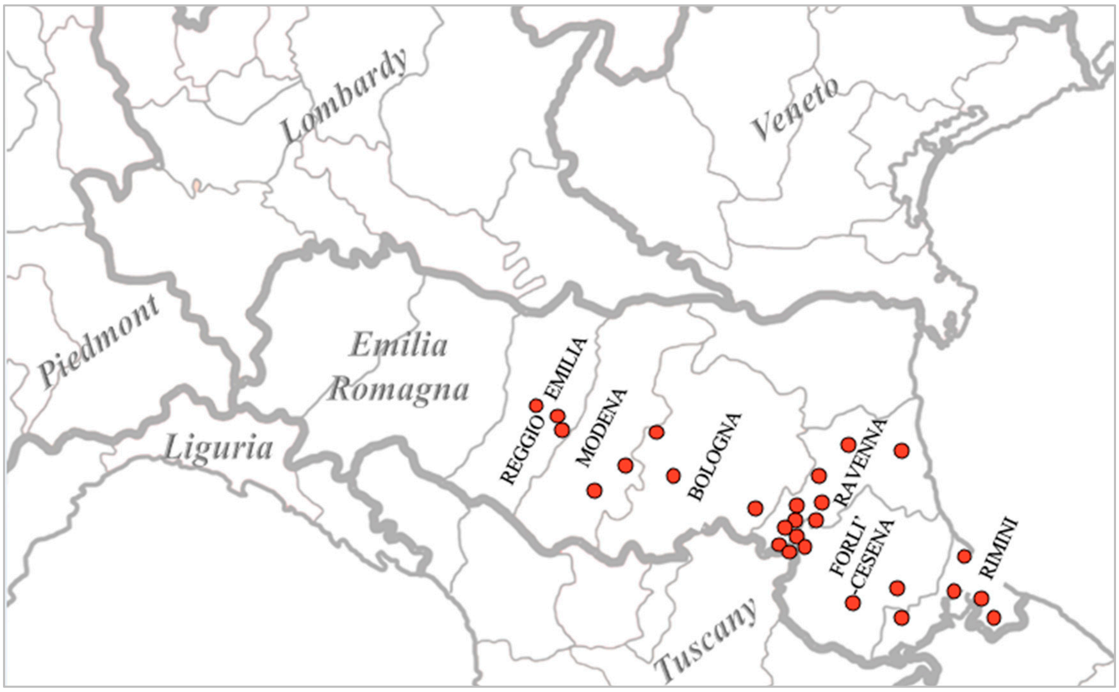

**Table S1.** Number of Mora Romagnola Herd Book registered farms, boars, sows and young pigs per year (from 2001 to 2019; [25]).

| Years | Farms | Boars | Sows | Young pigs[1] |
|---|---|---|---|---|
| 2001 | 17 | 24 | 41 | 155 |
| 2002 | 29 | 33 | 68 | 233 |
| 2003 | 43 | 52 | 103 | 411 |
| 2004 | 47 | 54 | 113 | 501 |
| 2005 | 47 | 67 | 129 | 684 |
| 2006 | 44 | 64 | 144 | 803 |
| 2007 | 48 | 77 | 177 | 989 |
| 2008 | 44 | 77 | 218 | 924 |
| 2009 | 42 | 82 | 218 | 660 |
| 2010 | 41 | 69 | 225 | 654 |
| 2011 | 44 | 69 | 234 | 675 |
| 2012 | 44 | 74 | 218 | 754 |
| 2013 | 44 | 66 | 234 | 658 |
| 2014 | 32 | 68 | 259 | 829 |
| 2015 | 34 | 68 | 277 | 1000 |
| 2016 | 33 | 77 | 320 | 1151 |
| 2017 | 34 | 64 | 275 | 1393 |
| 2018 | 33 | 70 | 314 | 1596 |
| 2019 | 33 | 66 | 311 | 1410 |

[1] Males that had less than 8 months of age or females until the first farrow.

**Table S2.** Number of Mora Romagnola pigs that were phenotyped in the years 2017-2019, distributed in different farms and provinces. The breeding animals were also genotyped.

| Province[1] | Farms[2] | Pigs per province | Breeding animals[3] | Young pigs[4] |
|---|---|---|---|---|
| Reggio Emilia | 3 | 25 | 25 (9 + 16) | 0 |
| Modena | 2 | 74 | 19 (4 + 15) | 55 |
| Bologna | 3 | 25 | 16 (6 + 10) | 9 |
| Ravenna | 12 | 530 | 222 (71 + 151) | 308 |
| Forlì Cesena | 2 | 19 | 18 (3 + 15) | 1 |
| Rimini | 5 | 153 | 57 (17 + 40) | 96 |
| Totals | 27 | 826 | 357 (110 +247) | 469 |

[1] Geographic distribution of the farms in the administrative units referred as provinces. Provinces are listed from west to east (see also Fig. S1). [2] Number of farms where pigs were phenotyped and sampled. [3] Total number of breeding pigs that were phenotyped and sampled for the subsequent genotyping. The first and second number in parenthesis indicates the boars and the sows. [4] Pigs with less than 6 months of age that were only phenotyped.

**Table S3.** Number of Mora Romagnola pigs having different phenotypes for three recorded exterior traits (coat colour, ear position and "*Linea sparta*").

| **Province**[1] | **No. of farms** | **Pigs per province** | **Coat colour**[2] | | | **Ears position**[4] | | | ***Linea sparta***[5] | |
|---|---|---|---|---|---|---|---|---|---|---|
| | | | **Standard** | **Red**[3] | **Other** | **Hanging** | **Half-hanging** | **Raised** | **Present** | **Absent** |
| Reggio Emilia | 3 | 25 | 25 | 0 | 0 | 25 | 0 | 0 | 25 | 0 |
| Modena | 2 | 74 | 55 | 19 | 0 | 73 | 0 | 1 | 70 | 4 |
| Bologna | 3 | 25 | 24 | 1 | 0 | 24 | 1 | 0 | 23 | 2 |
| Ravenna | 12 | 530 | 507 | 22 | 1 | 483 | 17 | 30 | 501 | 29 |
| Forlì Cesena | 2 | 19 | 19 | 0 | 0 | 19 | 0 | 0 | 19 | 0 |
| Rimini | 5 | 153 | 116 | 37 (1) | 0 | 151 | 2 | 0 | 146 | 7 |
| Totals | 27 | 826 | 746 | 79 | 1 | 775 | 20 | 31 | 784 | 42 |

[1] Geographic distribution of the farms in the administrative units referred as provinces. Provinces are listed from west to east (see also Fig. S1). [2]Recorded coat colour: Standard (black and tan in both adult and young pigs); Red (dark red over the whole body in adult or young pigs); Other coat colours in adult or young pigs (e.g. spotted patterns). [3] Red is considered a standard colour in the young pigs that have less than 6 months of age, according to the Herd Book of the breed; the red coat colour in the young pigs usually change to the black and tan colour in the adult age; the number of adult pigs out of all indicated pigs that still showed a red coat colour is reported within brackets (not considered the standard of the breed; adult animals with red coat colour are excluded from the Herd Book). [4]Hanging ears, ears that are bent forward and parallel to the muzzle constitute the standard of the breed; half-hanging and raised define other positions of the ears (half-hanging is intermediate between hanging and raised) that, in this study, have been observed only in young pigs. In adult animals, ears usually become hanging. If not, animals are excluded from the Herd Book of the breed. [5] In this study, the absence of "*Linea sparta*" was observed only in young pigs. Only pigs having this trait can be registered to the Herd Book of the breed.
